# Phosphorylation of the MBF Repressor Yox1p by the DNA Replication Checkpoint Keeps the G1/S Cell-Cycle Transcriptional Program Active

**DOI:** 10.1371/journal.pone.0017211

**Published:** 2011-02-16

**Authors:** Catia Caetano, Steffi Klier, Robertus A. M. de Bruin

**Affiliations:** MRC Laboratory for Molecular Cell Biology, University College London, London, United Kingdom; The National Institute of Diabetes and Digestive and Kidney Diseases, United States of America

## Abstract

**Background:**

In fission yeast *Schizosaccharomyces pombe* G1/S cell-cycle regulated transcription depends upon MBF. A negative feedback loop involving Nrm1p and Yox1p bound to MBF leads to transcriptional repression as cells exit G1 phase. However, activation of the DNA replication checkpoint response during S phase results in persistent expression of MBF-dependent genes.

**Methodology/Principal Findings:**

This report shows that Yox1p binding to MBF is Nrm1-dependent and that Yox1p and Nrm1p require each other to bind and repress MBF targets. In response to DNA replication stress both Yox1p and Nrm1p dissociate from MBF at promoters leading to de-repression of MBF targets. Inactivation of Yox1p is an essential part of the checkpoint response. Cds1p (human Chk2p) checkpoint protein kinase-dependent phosphorylation of Yox1p promotes its dissociation from the MBF transcription factor. We establish that phosphorylation of Yox1p at Ser114, Thr115 is required for maximal checkpoint-dependent activation of the G1/S cell-cycle transcriptional program.

**Conclusions/Significance:**

This study shows that checkpoint-dependent phosphorylation of Yox1p at Ser114, Thr115 results in de-repression of the MBF transcriptional program. The remodeling of the cell cycle transcriptional program by the DNA replication checkpoint is likely to comprise an important mechanism for the avoidance of genomic instability.

## Introduction

Cell proliferation of all organisms depends on the cell division cycle, which is initiated during the G1-phase of the cell cycle. Activation of a group of cell cycle-dependent transcripts in G1 initiates exit from G1 and entry into S-phase, thereby committing cells to a division cycle. In human cells G1-S transcription depends on the E2F transcription factor family, E2F1-8. Since E2F-dependent cell-cycle transcription is misregulated in nearly all tumor types, it is well studied. In the fission yeast *Schizosaccharomyces pombe*, this wave of transcription is largely dependent on one transcription factor complex named MBF (MluI cell cycle box (MCB) binding complex). MBF is composed of two homologous DNA-binding, zinc-finger proteins named Res1p [1], [2] and Res2p [3], [4], and the product encoded by the Start gene *cdc10+* [5], [6].

In fission yeast, MBF regulates the expression of at least 20 putative target genes. MBF-target promoters contain one or more MCB elements that serve as the platform for MBF binding. This cluster of MBF-target genes is enriched for genes encoding proteins involved in DNA synthesis, DNA repair and cell-cycle control [6], [7]. Well established MBF targets are the replication origin licensing factors *cdc18+* [8] and *cdt1+* [9] and the large subunit of the ribonucleotide-diphosphate reductase, *cdc22+* [6]. The molecular mechanisms involved in limiting expression of these genes to G1/S throughout normal cell cycle progression have been characterised extensively in fission yeast. The essential gene cdc10 encodes for a protein that is needed for MBF transcriptional activity [5], [6]. The Res1p and Res2p subunits are DNA binding proteins that generally play a positive and a negative regulatory role in MBF activity, respectively [4], [10], [11], [12], [13], [14]. However, apart from the subunits that comprise MBF, proper regulation of MBF-dependent transcription during the cell cycle requires additional co-regulators. Rep2p is a co-activator that is required for high levels of transcription but is not necessary for periodicity [10], [15], [16]. The transcriptional repressors Nrm1p and Yox1p are both involved in confining MBF-dependent transcription to the G1 phase of the cell cycle [17], [18], [19]. Nrm1p and Yox1p, involved in a negative feedback loop, are MBF targets themselves; they accumulate during S phase and bind to MBF at promoters and repress transcription outside of G1. The mechanism of MBF-dependent transcriptional activation during G1, and the role of Nrm1p and Yox1p in this process, remains largely unknown.

Once cells have committed to a division cycle they initiate DNA replication and progress into S-phase. In response to DNA damage or DNA replication stress cells activate the “DNA structure” checkpoints. The DNA structure checkpoints are required for the efficient response to genotoxic stress, which is critical for genome stability and cell survival. Whereas the DNA replication checkpoint is activated by replication fork arrest during S phase, the DNA damage checkpoint is activated in G2 phase when damaged DNA is detected. The mechanisms that halt cell cycle progression in the presence of incomplete DNA replication and DNA damage are mediated by an evolutionarily conserved subfamily of protein kinases [20], [21], [22], [23]. These include ATM and the closely related ATR in humans and Rad3 in fission yeast. These protein kinases exert their effect largely through the protein kinases Chk1 and Chk2 in mammals and Cds1 and Chk1 in fission yeast. In response to genotoxic stress the DNA structure checkpoints delay progression into mitosis to prevent chromosome segregation and to facilitate the appropriate response to the genomic stress. This response includes the induction of the transcription of genes that promote repair of cellular lesions including stabilization of stalled replication forks and induction of DNA repair functions.

In fission yeast the Cds1 protein kinase is activated primarily in response to stalled or collapsed DNA replication forks during S phase, whereas Chk1 is specifically activated in response to DNA damage outside of S phase [24], [25]. Persistent expression of MBF-dependent genes occurs in cells arrested in S phase with incompletely replicated DNA [26], which is dependent on functional Cds1 [14], [18], [27]. The current model for DNA replication stress-induced activation of MBF-dependent transcription involves the initial activation of Rad3p, which phosphorylates and activates Cds1, which in turn, phosphorylates Nrm1p, Cdc10p, and Ste9p, to keep MBF-dependent transcription active [14], [18], [28]. Whereas phosphorylation of Nrm1p and/or Cdc10p inhibits the binding of the corepressor Nrm1p to MBF at promoters, phosphorylation of Ste9p is thought to inhibit the transcriptional activator Rep2p being targeted for destruction by the Ste9/APC ubiquitin ligase complex.

Here we show that in response to DNA replication stress Yox1p is released from MBF promoters, which correlates with induction of MBF-dependent transcription. We show that phosphorylation of Yox1p at Ser114 and Thr115 by the DNA replication checkpoint protein kinase Cds1 is sufficient to keep MBF-dependent transcription active. Furthermore we establish that activation of MBF-dependent transcription is critical for cell survival in response to replicative stress.

## Results

### Yox1p binding to MBF is Nrm1-dependent

**Figure 1:**
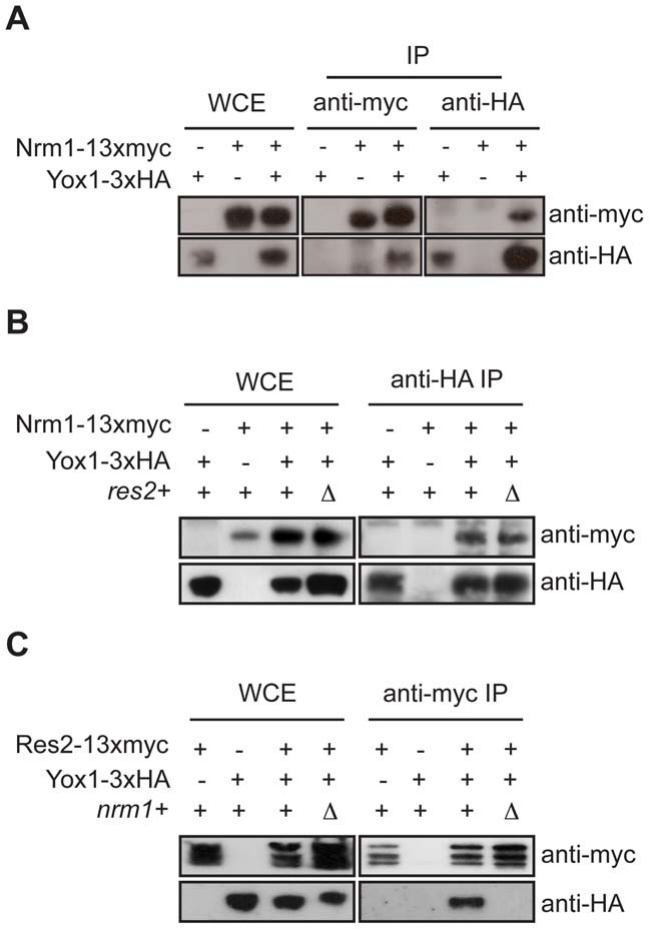
Yox1p interaction with MBF is Nrm1p-dependent. (A, B and C) Western blot analysis of anti-myc and anti-HA immune precipitates (IP) and whole cell extract (WCE), deriving from Nrm1-13xmyc, Yox1-3xHA, Nrm1-13xmyc-Yox1-3xHA and Res2-13xmyc tagged cells, in the presence or absence of either *nrm1+* or *res2+*. Tagged proteins were detected by anti-HA and anti-myc antibodies.

Recently, we have shown that Yox1p associates with the Cdc10p and Res2p components of the MBF complex [19]. To determine how Yox1 interacts with the MBF complex we carried out immunoprecipitations in strains carrying a deletion mutant of an untagged MBF component. We establish that Yox1p and Nrm1p are associated in wild type cells and inactivation of *res2^+^* does not abolish the interaction (Figure 1A and 1B). We have previously shown that Yox1p interacts with the MBF component Res2p [19]. However inactivation of *nrm1^+^* abolishes the interaction between Yox1p and Res2p (Figure 1C). Together, these results establish that Yox1p binding to MBF is Nrm1p-dependent.

### Yox1p and Nrm1p require each other for promoter binding

**Figure 2:**
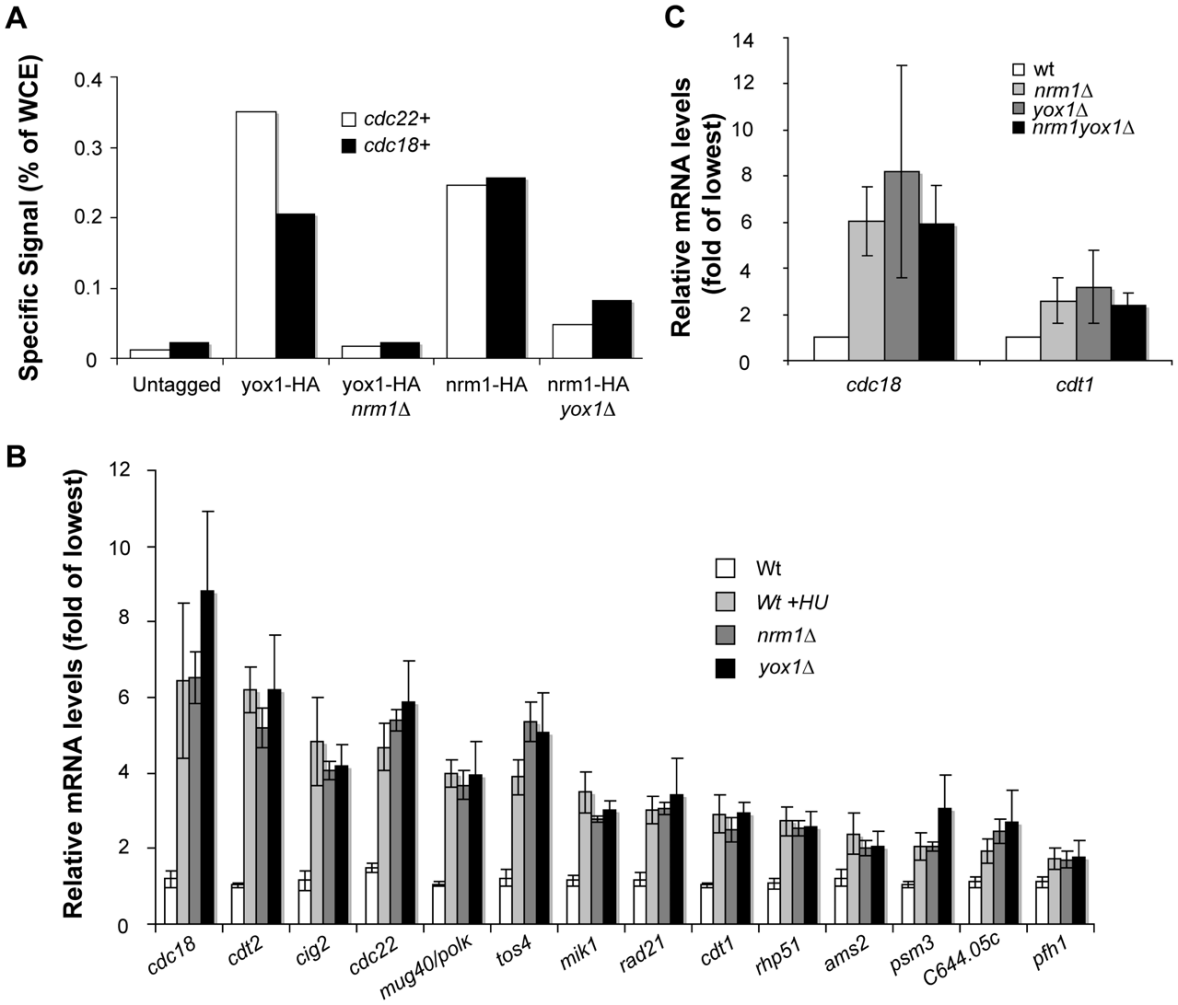
Yox1p and Nrm1 require each other to bind and repress MBF targets. (A) Chromatin-immune precipitated (ChIP) *cdc22* and *cdc18* promoter fragments pulled down by HA tagged Nrm1 and Yox1 in wild type, and *yox1Δ and nrm1*Δ cells, respectively, were quantified by qPCR. Enrichment is shown as percentage of WCE signal. Untagged cells were included as negative control and data shown are representative of multiple independent experiments (see Figure S1 for a biological repeat experiment). (B) Relative mRNA levels obtained by RT-qPCR for 14 MBF-dependent transcripts in untreated and HU treated wild type cells and *nrm1Δ* and *yox1Δ* cells. Transcript levels are shown as fold induction of transcript levels detected in wild type untreated cells. Bars represent the average value, and error bars represent their SD, obtained by qPCR of triplicate biological samples. (C) RT-PCR analysis of the relative levels of *cdc18*+ and *cdt1*+ transcripts in wild type, *nrm1Δ*, *yox1Δ* and *nrm1Δyox1Δ* cells in untreated conditions and as percentage of maximal levels (100%). Bars represent the average value, and error bars represent their SD, obtained by qPCR of triplicate biological samples.

These results are consistent with our previous observation that Yox1p binding to MBF promoters depends on Nrm1p [19]. Like Yox1p, binding of Nrm1 to MBF target promoters depends upon its DNA binding component Res2 [18], [19]. This is despite the capacity of Nrm1p and Yox1p to bind each other without Res2 (Figure 1B and 1C). To assess whether Nrm1p requires Yox1p to bind MBF at MBF-dependent promoters we carried out chromatin Immunoprecipitation experiments (ChIP). As previously demonstrated, Nrm1p and Yox1p bind efficiently to the MBF targets, *cdc22+* and *cdc18+* [18], [19]. As previously observed Yox1p does not bind detectably to these promoters in *nrm1*Δ cells, and our data shows that binding of Nrm1p to these promoters is also significantly reduced in a *yox1Δ* strain (Figure 2A and S1). The partial binding of Nrm1p to MBF-regulated genes in the absence of Yox1p, and complete loss of binding of Yox1p in *nrm1*Δ cells suggests that both proteins require each other for proper binding to MBF.

### Yox1p and Nrm1p are both required to repress MBF-dependent transcription

Consistent with their binding dependency both Nrm1p and Yox1p are required for repression of MBF-regulated transcription outside of G1 [18], [19]. To determine the contribution of both proteins to the repression of MBF-dependent transcription we analysed the expression levels of 14 MBF-dependent transcripts in wt, *nrm1Δ* and *yox1Δ* cells (Figure 2B). Consistent with data obtained previously from microarray expression profiling [19], deletion of *yox1+* promotes an overall upregulation of the MBF transcriptional program. The fold-induction generated by abrogation of *yox1+* varies widely across the studied transcripts with a maximum of 7.4-fold for *cdc18+* and a minimum of 1.6-fold for *pfh1+* (Figure 2B). The expression signature of the same transcripts in *nrm1Δ* cells is similar, if not identical, to that observed for *yox1Δ* cells. Furthermore, inactivation of both *yox1*+ and *nrm1*+ does not significantly increase transcript levels compared to the single mutants (Figure 2C). These data indicate that their mutual dependency for proper promoter binding is likely to be the cause for their non-redundant role in transcriptional repression. Alternatively, the residual binding of Nrm1p to MBF target promoters in *yox1Δ* cells could indicate that Nrm1p serves mainly as a scaffold for binding of Yox1p to MBF. Overall, our data shows that both Nrm1p and Yox1p are required to repress MBF-dependent transcription.

### Yox1p and Nrm1p dissociate from promoters in response to DNA replication stress

**Figure 3:**
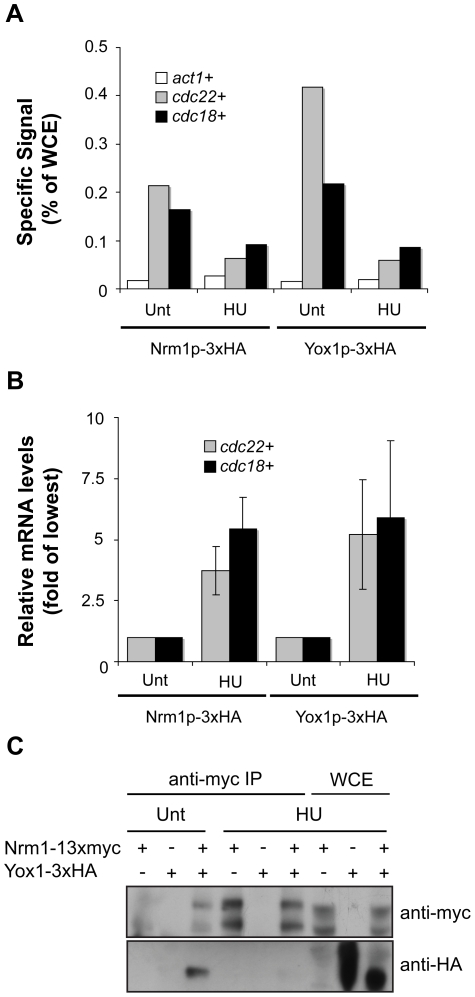
Yox1p is unable to bind and repress transcription in response to DNA replication stress. (A) Promoter fragments from Nrm1p-HA and Yox1p-HA ChIPs were quantified using qPCR from untreated and HU treated cells. Bar graphs represent percentage of WCE signal. Data representative of multiple independent experiments (see Figure S2 for a biological repeat experiment). (B) RT qPCR analysis on RNA isolated levels from untreated and HU-treated cells before cross-linking, are shown as fold of lowest relative levels detected. Error bars represent the SD of three independent biological repeats. (C) SDS PAGE analysis of anti-HA and anti-myc IPs and WCE for proteins deriving from same cells.

Part of the DNA replication transcriptional response is to maintain MBF-dependent transcription at a high level [14], [18], [27], [28], [29], [30]. The level of transcription observed in HU treated cells is comparable to levels observed in both *yox1*Δ and *nrm1*Δ cells indicating that Yox1p/Nrm1-dependent repression is inactivated (Figure 2B). Based on these data we hypothesise that Yox1p could represent an additional target of the DNA replication checkpoint to keep MBF transcription active. To test this we carried out ChIP analysis on Yox1p and Nrm1p in untreated and HU treated cells, and measured the transcript levels of the *cdc18*+ and *cdc22*+ MBF-targets. As observed for Nrm1p, HU-induced DNA replication checkpoint activation results in loss of Yox1p from the *cdc22+* and *cdc18+* promoters (Figure 3A and S2), with a corresponded increase in the mRNA levels of the same genes (Figure 3B). Since Nrm1p leaves promoters in response to HU treatment and Yox1p binding to MBF at promoters depends on Nrm1p these results are not surprising.

To establish whether Nrm1p and Yox1p stay in complex in response to HU treatment we carried out immunoprecipitation in untreated and HU treated cells expressing Nrm1p-myc Yox1p-HA. This reveals that activation of the DNA replication checkpoint abrogates the interaction between Yox1p and Nrm1p (Figure 3C). Together, these lines of evidence suggest that both Yox1p and Nrm1p dissociate from MBF at promoters in response to activation of the DNA replication checkpoint.

### Yox1p phosphorylation is dependent upon the checkpoint protein kinases

**Figure 4:**
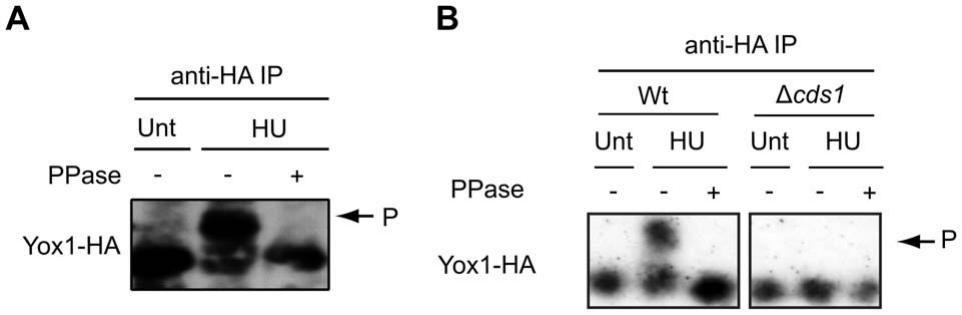
Yox1p HU-induced phosphorylation is Cds1p dependent. (A and B) Yox1-HA in WCE and HA-enriched lysates deriving from untreated and HU-treated cells detected by high-affinity anti-HA antibody.

It has been shown that Nrm1p is phosphorylated in a checkpoint dependent manner following HU treatment [18]. Furthermore, phosphorylation of the C-terminal region of Cdc10 has also been implicated in the mechanism by which the checkpoint activates MBF dependent transcription [14]. To establish whether Yox1p is phosphorylated in response to HU treatment we monitored Yox1p migration by Western blot analysis from untreated and HU-treated Yox1-HA cell lysates. As shown for Nrm1p [18], treatment with HU results in accumulation of a series of higher molecular weight species of Yox1p-HA that migrate slower in the SDS-polyacrylamide matrix compared to Yox1p-HA from untreated cells (Figure 4A and 4B). To test if the slower migrating species of Yox1p-HA present in HU-treated samples are the result of phosphorylation, immunoprecipitated Yox1-HA was treated with λ-phosphatase. This shows that phosphatase treatment collapses the slower migrating species (Figure 4A and 4B) and that Yox1p, like Nrm1p, is phosphorylated in response to checkpoint activation. Given the involvement of Cds1p in the phosphorylation and inactivation of Nrm1p [18], we sought to determine whether phosphorylation of Yox1p in response to HU is also Cds1-dependent. Analysis of Yox1p-HA mobility in *Δcds1* cells after HU treatment reveals that the phospho-shift is impaired in the absence of Cds1p, (Figure 4B). Hence, Yox1p phosphorylation in response to HU treatment is Cds1-dependent.

### Inactivation of Yox1p is an essential part of the checkpoint response

**Figure 5:**
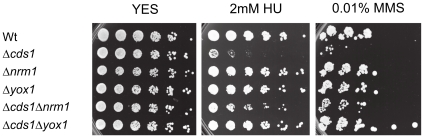
Inactivation of Yox1 following genotoxic stress is essential for cell survival. Five-fold serially dilution of wt, *cds1Δ*, *Δnrm1*, *yox1Δ*, *cds1Δnrm1Δ* and *cds1Δyox1Δ* cells were spotted onto YES or YES plus indicated concentrations of HU or MMS.

*Cds1* null mutant cells are extremely sensitive to the deleterious effects caused by HU and methyl methane sulfonate (MMS) [31]. This is attributable, in part, to their inability to maintain the MBF transcriptional program [18], [32]. Constitutive activation of MBF-dependent transcription as observed in *nrm1*Δ cells suppresses the sensitivity of *cds1*Δ cells to chronic exposure but does not seem to have a role in the acute response to genotoxic stress [30]. Based on this, we hypothesised that deletion of *yox1+*, would suppress sensitivity of *cds1*Δ cells to HU and MMS. To test this hypothesis we compared the sensitivities of wt, *Δcds1*, *Δnrm1*, *Δyox1*, *Δcds1Δnrm1* and *Δcds1Δyox1* cells to chronic exposure to HU and MMS, through the application of survival assays. The obtained results revealed that, like *nrm1*Δ, deletion of *yox1+* suppresses the sensitivity of *Δcds1* cells to both HU and MMS (Figure 5). In this context, failure to inactivate Yox1p due to absence of Cds1p is rescued by abrogation of Yox1p itself demonstrating that inactivation of Yox1p is a vital step in the checkpoint response.

### Phosphorylation of Yox1p S114, T115 sites play an important role in checkpoint regulation of MBF transcription

**Figure 6:**
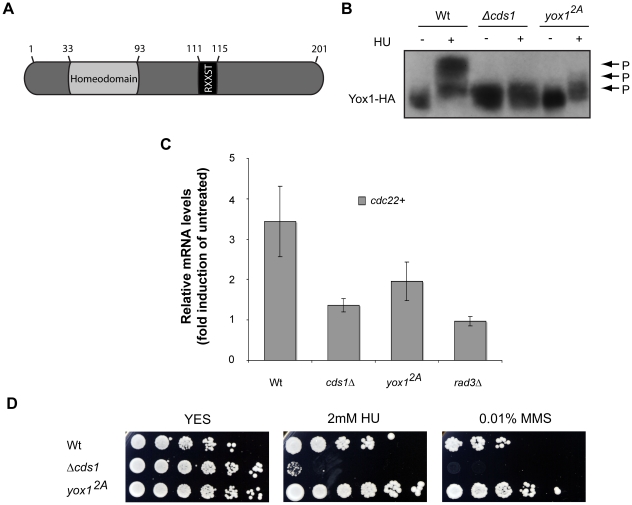
HU challenge induces phosphorylation of Yox1 at its RXXST consensus. (A) Cartoon diagram displaying the molecular arrangement of the homeodomain and the putative RXXS/T motif in Yox1p. Not to scale. (B) SDS-PAGE electrophoresis of Ha tagged Yox1 in untreated and HU-treated wt, *Δcds1*, and *yox1^2A^* cells as described before. (C) RT-PCR analysis of *cdc22+* transcript levels corresponding to fold induction over untreated wt for the same cells as in B and in *rad3Δ* cells before formaldehyde-induced cross-linking. Bars represent the average value, and error bars represent their SD, obtained by qPCR of triplicate biological samples. (D) Five-fold serially dilution volumes of wt, *yox1^2A^* and *cds1Δ*, cells were spotted onto YES or YES plus indicated concentrations of HU or MMS.

Since Nrm1p has been shown to be a direct target of Cds1 *in vitro* [18] we hypothesized that phosphorylation of Yox1p by Cds1 might promote its dissociation from MBF. In an effort to establish the requirement for Yox1p phosphorylation for its release from the transcription complex, we looked for the putative Cds1p-recognition motif, RXXST [33], [34], [35], in the Yox1p amino-acid sequence. We identified one such consensus sequence at amino-acids 111-115 of Yox1p (Figure 6A; RRKST). Conversion of the Ser114 and Thr115 sites to alanine at the endogenous locus, creating the *yox1^2A^* mutant strain, results in a dramatic effect on the mobility of the mutant protein in response to HU *in vivo* (Figure 6B). This indicates that this is one of the mains sites that is phosphorylated in a checkpoint-dependent manner. Consistent with a possible role in phosphorylation-dependent inactivation of Yox1 by Cds1, we observe significant repression of MBF targets *cdc18*+ and *cdc22*+ in response to HU treatment in the *yox1^2A^* mutant (Figure 6C). The level of expression is significantly lower than that observed in wild-type cells but somewhat higher than that observed in the *cds1*Δ and *rad3*Δ checkpoint mutants. The inability to fully induce MBF-dependent transcription in response to checkpoint activation in the *yox1^2A^* mutant does not result in an increase in HU sensitivity (Figure 6D). These results are consistent with a significant contribution of Ser114, Thr115 phosphorylation in the checkpoint-dependent regulation of Yox1p activity in response to HU treatment.

## Discussion

**Figure 7:**
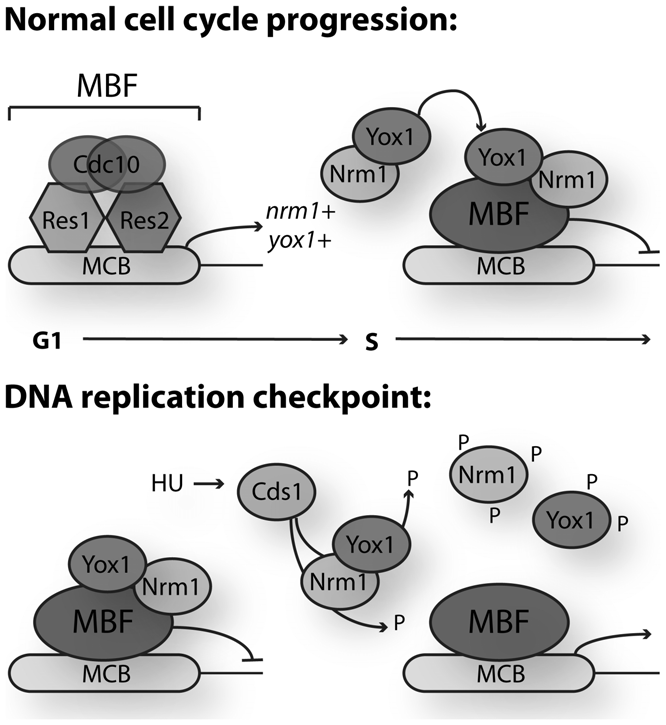
Regulation of Yox1p during the cell cycle and in response to DNA replication stress. Inactivation of MBF-dependent transcription during late S phase of the normal cell cycle is dependent on binding of the co-repressor Yox1p through Nrm1p to the core components of the MBF transcription factor (upper panel). Activation of the DNA replication checkpoint and maintenance of the MBF transcriptional program following HU challenge occurs via phosphorylation and inactivation of both Nrm1p and Yox1p by the DNA replication checkpoint effector kinase Cds1 (lower panel).

In fission yeast, *Schizosaccharomyces pombe*, a negative feedback loop involving Nrm1p and Yox1p bound to MBF, represses G1/S cell-cycle regulated transcription, once cells progress into S phase [17], [19]. In response to loss of integrity of the DNA replication fork, cells activate the DNA replication checkpoint. Part of the DNA replication checkpoint transcriptional response is to maintain MBF-dependent transcription at a high level of persistent expression [14], [18], [27], [28], [29], [30]. In this report we show that in response to DNA replication stress both Yox1p and Nrm1p dissociate from MBF promoters, leading to de-repression of MBF targets (figure 7). Inactivation of either Yox1 or Nrm1 in a checkpoint mutant background significantly suppresses the sensitivity of those cells to genotoxic agents such as HU or MMS. This indicates that de-repression of MBF-dependent transcripts is vital for viability of cells in response to genotoxic stress. We show that mutating one putative Cds1 site in Yox1p results in significant repression of MBF targets during a DNA replication checkpoint response. This suggests that phosphorylation of this single site is important to de-repress transcription. The *yox1^2A^* mutant does not display an increase in HU sensitivity. It seems likely that stronger interference with checkpoint-dependent de-repression of MBF transcription would be required to establish its importance. Previous reports suggest that phosphorylation of Nrm1p and/or Cdc10p inhibits the binding of the corepressor Nrm1p to MBF at promoters. However, mutating several potential phospho sites in either Nrm1 and/or Cdc10 did not result in complete loss of induction of MBF target genes in response to DNA replication stress [14], [18]. We speculate that it might require a triple Yox1p, Nrm1p, Cdc10p phospho-site mutant to completely abrogate the checkpoint-dependent activation of the G1/S cell-cycle transcriptional program. It will be important to investigate whether reduced levels of MBF-dependent transcription during a checkpoint response as observed in our Yox1^2A^ mutant affects genome stability. Overall further research is required to establish whether full repression of MBF-dependent transcription during DNA replication stress is detrimental to cells.

Yox1 and Nrm1 are involved in a negative feedback loop to confine G1/S transcription to the G1-phase of the cell cycle. Genetic perturbation of either Yox1 or Nrm1 leads to increased MBF-dependent transcription indicating that both proteins are required, but are not sufficient, to repress MBF transcription outside of G1 phase. One can argue that this creates a less robust system to repress transcription, since mutations that affect either Yox1 or Nrm1 will result in loss of cell cycle regulated transcription. Based on the same argument the use of two non-redundant proteins creates a more robust regulatory system when MBF-dependent transcription needs to be de-repressed outside of G1. So why use two non-redundant proteins to repress transcription during the cell cycle? Here we show that in response to DNA replication stress the DNA replication checkpoint de-represses MBF-dependent transcription by releasing both Yox1 and Nrm1 from MBF at promoters. Moreover, inactivation of either Yox1 or Nrm1 in a *cds1*Δ checkpoint mutant largely rescues the sensitivity of these cells to HU, indicating that de-repression of MBF-dependent transcription is important for viability in response to genotoxic stress. So whereas confining MBF-dependent transcription to the G1 phase of the cell cycle is not essential in rapidly growing cells as *nrm1Δ*, and *yox1Δ* deletion mutants are viable we show that de-repression of MBF-dependent transcription is essential in response to genotoxic stress. Overall the requirement for these multiple, non-redundant transcriptional repressors is striking and may reflect the importance of timely de-repression in response to genotoxic stress over robust down-regulation of MBF target genes once cells proceed through S-phase.

Our study shows that Yox1p plays a central role in the mechanism by which the DNA replication checkpoint maintains high levels of G1/S transcription in response to DNA replication stress. Many G1/S genes encode proteins required for DNA replication and repair. It is therefore thought that accumulation of G1/S transcripts might be important for reinitiation of stalled replication forks and for the restoration of robust DNA replication following a DNA replication block. In humans G1/S gene expression depends on the E2F family of transcription factors and their regulators, the pRb family members. Whereas these proteins have no recognizable sequence homology with their yeast counterparts, they play analogous roles in their respective systems. Interestingly the DNA damage transcriptional response in humans includes the regulation of many genes that encode for proteins involved in DNA replication and repair of DNA damage many of which are regulated by the E2F family of transcription factors during the G1/S transition. This suggests that regulation of G1/S transcription by the DNA replication checkpoint, as shown in fission yeast, may also be conserved in humans [29]. As putative targets of cell cycle checkpoints that regulate genomic stability, the G1/S transcription factors and their regulators are expected to play a central role in the avoidance of DNA damage and chromosomal aberrations, phenomena that directly contribute to tumorigenesis. Consequently, understanding the mechanisms governing regulation of G1/S gene expression in response to genotoxic stress may provide new insights into the genesis and treatment of human cancer.

## Materials and Methods

### Yeast strains, experimental conditions and phenotyping

The *nrm1Δ* and *yox1Δ* mutants and the *res2*-13xmyc, *nrm1*-13xmyc, *nrm1-*3xHA, and *yox1-*3xHA C-terminal 3xHA-tagged strains are described previously [18], [19]. The *yox1^2A^-*3xH*A* mutants carrying amino acid substitutions S114A and T115A, were generated by PCR using the Quick-Change XL site-directed mutagenesis strategy (Stratagene). Yox1^2A^ -3xHA was integrated at the endogenous locus via homologous recombination and mutations confirmed by DNA sequencing. All strains were grown in rich medium (YE+supplements) at 30°C. DNA replication stress was induced by treating cells with HU (12 mM) for 3.5 h at 30°C. See Table S1 for strains list.

### Co-immunoprecipitation and SDS-PAGE

For each IP, 50 ml of exponentially growing cells were mechanically disrupted (FastPrep) in lysis buffer containing protease inhibitors (Complete Mini, Roche) and phosphatase inhibitors (Phosphatase Inhibitor Cocktail 1, Sigma-Aldrich) and glass beads (BioSpec) by 4×30 s cycles with 4 minutes cool down periods. Subsequently Nrm1p-myc, Nrm1p-HA and Yox1p-HA were immunoprecipitated with either anti-HA (12CA5, Roche) or anti-myc (9E10, Santa Cruz Biotechnology) antibodies, by incubating lysates for 2 h at 4°C with 50 ul of 50% protein A Sepharose beads. SDS sample buffer was added to protein purified on beads and resolved by 10% SDS-PAGE. Nrm1p-myc was detected using the previously described antibody, and Nrm1p-HA and Yox1p-HA a high affinity anti-HA (3F10, Roche) antibody.

### Phosphatase treatment assay

Lysates deriving from 50 ml of exponentially growing cells were enriched for Yox1p-HA as indicated above. Bead bound protein was washed 3× in IP buffer containing protease but not phosphatase inhibitors, resuspended in 900 ul of washing buffer, divided into 3 and treated with either IP buffer alone, and IP buffer plus active λ-protein-phosphatase (1200 units final concentration, Sigma). Samples were then allowed to incubate at room temperature for 30 min, disrupted in SDS sample buffer and resolved in 10% SDS-PAGE as described previously.

### ChIP analysis

ChIP analysis was carried out as decribed in Aligianni *et al* [19]. In summary, 45 ml of exponentially growing cells were treated with formaldehyde (37% v/v) for 30 min, to 1% final concentration for DNA-protein crosslinking. Crosslinking reaction was then stopped by adding glycine (2.5 M) to a final concentration 125 mM. Pelleted cells were washed 3 times with cold TBS, resuspended in 500 µl lysis buffer complemented with protease and phosphatase inhibitors and disrupted as described before. Resulting chromatin fractions were subsequently resuspended in fresh lysis buffer, sonicated in a Bioruptor (Diagenode) for a total time of 30 min (30 sec ON, 5 min OFF) and immunoprecipitated with anti-HA antibody (12CA5, Roche) overnight, plus 50 µl 50% PAS for four more hours. Protein-DNA-bead complexes were washed 2 times in lysis buffer (no inhibitors), 2 times in lysis buffer containing NaCl (360 mM), 2 times in wash buffer and 1 time in TE buffer, for 15 min in each individual solution. Washed complexes were incubated in 100 ul of elution buffer for 30 min at 65°C and resulting supernatants and previously prepared WCEs further incubated at 65°C overnight to reverse crosslinking. Finally, samples were purified and quantified using the Qiaquick PCR Purification (Qiagen). The iQ SYBR Green supermix (Bio-Rad) kit was used in RT-PCR reactions run on a Chromo-4 Real-Time PCR Detector (Bio-Rad). Data was analysed using MJ Opticon Analysis Software 3.0.

### Reverse transcriptase (RT) quantitative (q)PCR

Total RNA was prepared using the RNeasy Plus Kit (Qiagen) as indicated in the manufacturer's manual. Transcript levels were determined by RT qPCR using the iScript One-Step RT-PCR kit with SYBR Green Supermix (Bio-Rad). Data was analysed as described above.

### Spot Assays

Cells were grown in YES to OD_600_ 0.6. Cultures were 5-fold serially diluted and spotted on drug-free and HU (2 mM)- and MMS (0.01%)-containing YES plates using a purpose-built, replica-pin apparatus. Plates were incubated for at least four days at 30°C and pictures taken using an Epson Expression 1680 Pro scanner.

## Supporting Information

Figure S1 Biological repeat experiment of Figure 2A displaying occupancy of Yox1-HA in the MBF targets *cdc22+* and *cdc18+*. For description refer to Figure 2A legend.
(TIF)

Figure S2 Biological repeat experiment of Figure 3A. See Figure 3A legend for experimental details.
(TIF)

Table S1 Strains used in this study.
(DOCX)
