# Supplementary figures and images for: Phosphorylation of the MBF Repressor Yox1p by the DNA Replication Checkpoint Keeps the G1/S Cell-Cycle Transcriptional Program Active
Source: PLoS One. 2011 Feb 16;6(2):e17211. doi: 10.1371/journal.pone.0017211 (PMC3040222; doi:10.1371/journal.pone.0017211)

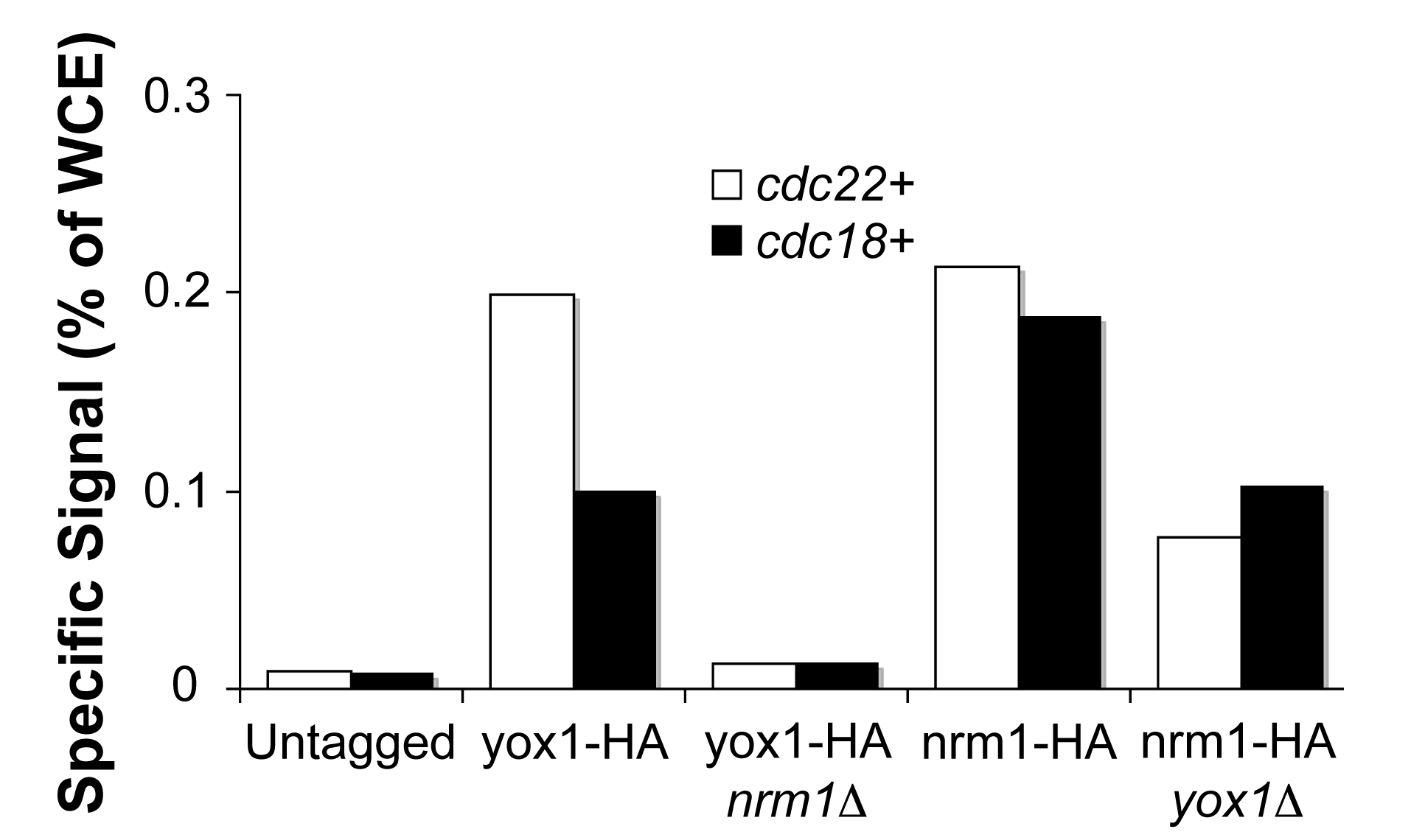

Supplement: Figure S1 — Biological repeat experiment of Figure 2A displaying occupancy of Yox1-HA in the MBF targets cdc22+ and cdc18+. For description refer to Figure 2A legend. (TIF) [file pone.0017211.s001.tif]

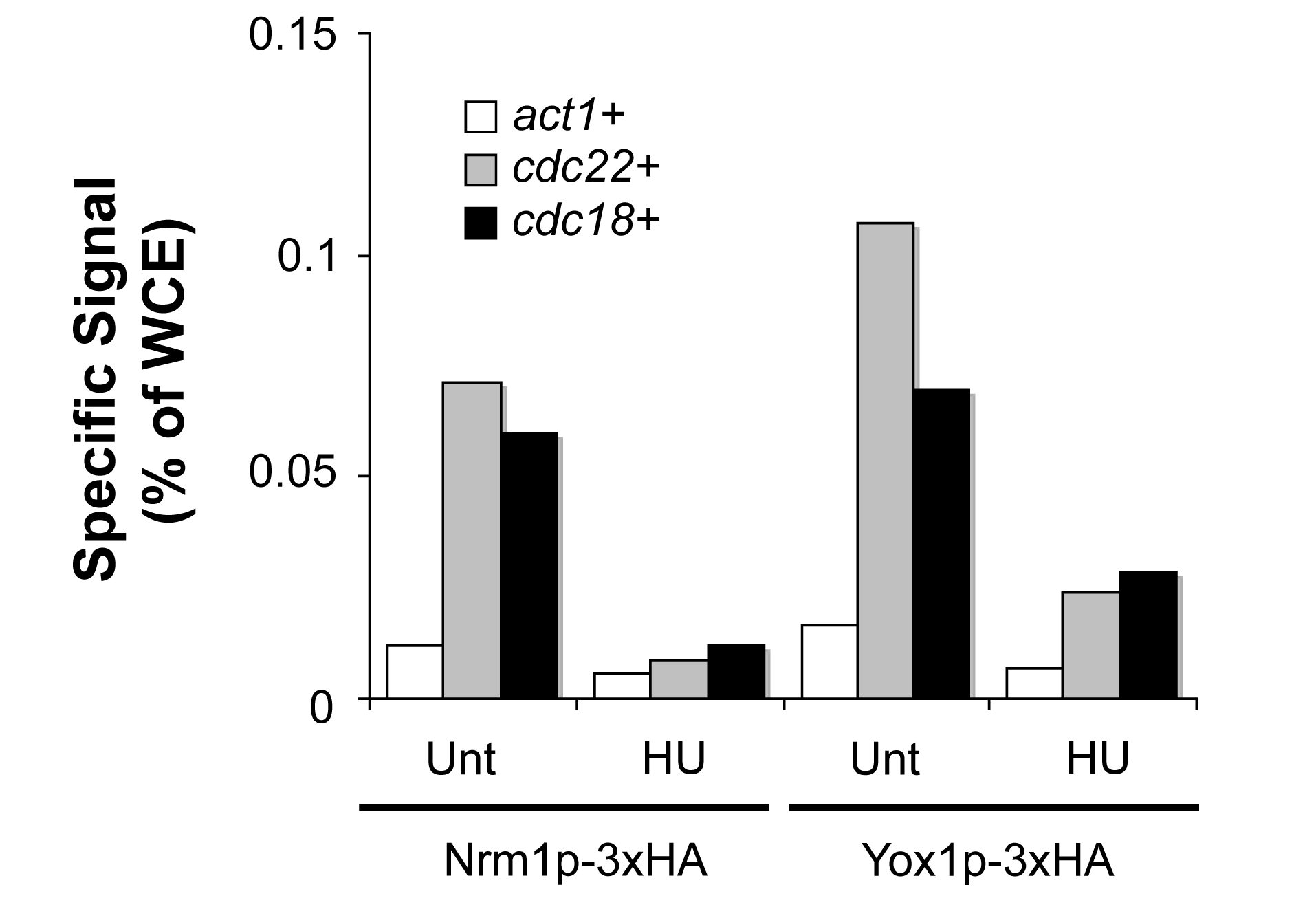

Supplement: Figure S2 — Biological repeat experiment of Figure 3A. See Figure 3A legend for experimental details. (TIF) [file pone.0017211.s002.tif]
