# Supplementary material for: Phosphorylation of the MBF Repressor Yox1p by the DNA Replication Checkpoint Keeps the G1/S Cell-Cycle Transcriptional Program Active
Source: PLoS One. 2011 Feb 16;6(2):e17211. doi: 10.1371/journal.pone.0017211 (PMC3040222; doi:10.1371/journal.pone.0017211)
Supplement: Table S1 — Strains used in this study. (DOCX) [file pone.0017211.s003.docx]

**Table 1**

Strain Genotype Source

RBP7 *h^+^ leu-32 ura4-D18*

RBP12 *h^+^* nrm1-13xmyc::kan this study

RB386 *h^-^ yox1-3xHA::kan* (Aligianni *et. al*., 2009)

RBP388 *nrm1-13xmyc::kan-yox1-3xHA::kan* this study

RBP390 *h^+^ nrm1-13xmyc::kan-res2Δ::ura4*  (de Bruin *et. al*., 2006)

RBP398 *yox1-3xHA::kan-res2Δ::ura4*  (Aligianni *et. al*., 2009)

RBP402 *nrm1-13xmyc::kan-yox1-3xHA::kan-res2Δ::ura4* this study

RBP13 *h^-^ res2-13xmyc::kan* (de Bruin *et. al*., 2006)

RBP398 *res2-13xmyc::kan-yox1-3xHA::kan-nrm1Δ::hyg* this study

RBP6 *h^+^ nrm1-3xHA::kan* (de Bruin *et. al*., 2006)

RBP400 *nrm1Δ::hyg-yox1-3xHA::kan*  this study

RBP419 *yox1Δ::kan-nrm1-3xHA::kan* this study

RBP425 *cds1Δ::ura4-yox1-3xHA::kan* this study

RBP449 *h^+^ yox1^2A^-3xHA::kan* this study

RBP35 *h^-^ cds1Δ::ura4* (Boddy *et. al*., 1998)

RBP8 *h^+^ nrm1Δ::kan* (de Bruin *et. al*., 2006)

RBP387 *h^+^* *yox1Δ::kan* (Aligianni *et. al*., 2009)

RBP70 *cds1Δ::ura4-nrm1Δ::kan* (de Bruin *et. al*., 2008)

RBP406 *cds1Δ*::*ura4*-*yox1Δ*::*kan* this study

RBP77 *h^-^ rad3*∆::*ura4* (Rhind *et. al*., 1998)

RBP419 *nrm1Δ::kan yox1Δ::kan* this study
